# Physiotherapy students’ knowledge and attitudes about their role in mental health

**DOI:** 10.4102/sajp.v79i1.1867

**Published:** 2023-07-11

**Authors:** Ropafadzo R. Gunduza, Sandy Lord, Monique M. Keller

**Affiliations:** 1 Department of Physiotherapy, Faculty of Health Sciences, School of Therapeutic Sciences, University of the Witwatersrand, Johannesburg, South Africa

**Keywords:** mental health, mental illness, knowledge, attitudes, physiotherapy, physiotherapy students

## Abstract

**Background:**

As the prevalence of mental health disorders (MHD) increases, physiotherapy students will be increasingly exposed to patients with MHD in their undergraduate studies. International research has shown that students who have mental health included in their curriculum have more knowledge and positive attitudes towards patients with MHD. In South Africa, little is known about physiotherapy students’ attitudes towards and knowledge of mental health.

**Objectives:**

To establish the knowledge of and attitudes towards the role of physiotherapy in determining the mental health attitudes.

**Method:**

In our cross-sectional, descriptive study, third- and fourth-year physiotherapy students at the University of the Witwatersrand were purposefully sampled. Online questionnaires, based on the Mental Health Knowledge Schedule (MAKS) and Mental Illness Clinicians’ Attitudes (MICA) scales were completed.

**Results:**

Thirty-four students participated in our study. Overall, all students indicated a moderate level of knowledge and a positive attitude towards mental health with mean MAKS score: 26.94 (standard deviation [s.d.]: 3.57) and MICA-4 score: 39.12 (s.d.: 16.12). Fourth-year students had more knowledge and a better attitude towards mental health when compared to the third-year students.

**Conclusion:**

Third- and fourth-year University of the Witwatersrand physiotherapy students have moderate knowledge and a positive attitude towards the role of physiotherapy in mental health.

**Clinical implications:**

The inclusion of mental health in the physiotherapy curriculum may improve students’ knowledge and attitudes towards mental health and prepare them for managing these complex patients in the future.

## Introduction

The prevalence of mental health disorders (MHD) has increased over the past 20 years. In 2017, 970 million people were globally diagnosed with MHD, contributing 5% – 10% of the global burden of disease (Ritchie & Roser 2020; Sankoh, Sevalie & Weston 2018). In Africa, between 2000 and 2015, the number of years lost to disability because of MHD and substance abuse disorders increased by 52% (Sankoh et al. 2018). The World Health Organization (WHO) states that the suicide mortality rate in South Africa as of 2019 was 23.5% per 100 000 population (WHO 2019).

Poor mental health can negatively impact physical health, and the risk of developing MHD can be exacerbated by poor physical health (Connaughton & Gibson 2016a). According to the World Health Organization (WHO), people with severe MHD, for example, schizophrenia and major affective disorders such as depression and bipolar disorder, have a general life expectancy of 10–20 years less than the general population (Arnoldy, Curtis & Samaras 2014; Lee et al. 2017; WHO 2020). These premature deaths can often be attributed to poor physical health conditions that are comorbid with MHD (Connaughton & Gibson 2016a). These include cardiovascular disease, ischaemic heart disease, hypertension, diabetes and respiratory disease (Connaughton & Gibson 2016a).

Physiotherapists are members of the multidisciplinary health care team (MDT) and play an integral role in patient education related to mental health and referring further for specialised care where indicated. Physiotherapists also promote function and overall well-being using evidence-based approaches including physical activity, exercise, movement, relaxation techniques and body and movement awareness (Andrew et al. 2019; IOPTMH 2019; Probst 2017; Stubbs et al. 2014). Despite studies published worldwide, the physiotherapist’s role in mental health is seldom appreciated (Probst 2017). This is because of physiotherapists being under-utilised in the treatment of people with MHD (Lee et al. 2017). For example, in Australia, physiotherapy is neither considered a primary profession in the mental health work force nor recognised as playing a vital role in delivering services to mental health patients (Connaughton & Gibson 2016b). Physiotherapists in the United Kingdom (UK) have also expressed a lack of education and experience within mental health specialities and the effect that has on managing patients’ psychosocial aspects of care (Hemmings & Soundy 2021).

Physiotherapy students have both the knowledge and skills to manage musculoskeletal (including chronic pain), cardiopulmonary and neurological disorders. However, little training is provided regarding addressing comorbid mental health issues that are often present in these patients (Connaughton & Gibson 2016a). According to Probst and Peuskens (2010) some physiotherapy curricula show little interest in mental health and psychiatry; thus, physiotherapists lack the opportunity to develop the necessary skills to manage individuals with MHD. Ntsiea et al. (2017) found that the physiotherapy curriculum adequately included all the medical conditions seen as a primary diagnosis by physiotherapists in all Gauteng public hospitals. The study mentioned that ‘stress’ as a primary diagnosis comprised only 0.3% of the total types of conditions seen and as such was not specifically covered in the curriculum. However, most patients present with multi-morbidities and as a result, stress and other MHD may be more prevalent and are not being included in the management of many patients.

As the prevalence of MHD increases globally, physio-therapists and physiotherapy students will increasingly be exposed to patients with MHD. The evidence shows that physiotherapists play an important role in the management of patients with MHD. As such more knowledge and a positive attitude are needed to confidently manage these patients (Probst 2017, Vancampfort et al. 2018). The more knowledge that physiotherapy students have about MHD, the better their attitudes towards patient care (Probst and Peuskens 2010). Our study therefore aimed to determine the knowledge of and attitudes towards the role of physiotherapy in MH of physiotherapy students.

## Methods

A quantitative, descriptive cross-sectional design with an online survey was used in our study. Participants included third- and fourth-year undergraduate physiotherapy students from the Physiotherapy Department at the University of the Witwatersrand during 2021. We included only third- and fourth-year physiotherapy students because students would have been exposed to clinical work by the third year and possibly encountered patients with comorbid MHD. Purposive sampling was used for participant selection. Surveys were sent to all third- and fourth-year physiotherapy students through the Research Electronic Data Capture (REDCap) online platform and the responses were returned anonymously. Regular emails to remind students to participate and invitations to participate were sent through other digital communication platforms. The REDCap link included the participation information sheet and consent form, a demographic survey and the Mental Health Knowledge Schedule (MAKS) and Mental Illness Clinicians Attitudes version 4 (MICA-4) scales.

### Data collection tools

The primary outcomes of our study were knowledge of and attitudes towards the role of physiotherapy in determining mental health attitudes. The MAKS questionnaire was used to assess the knowledge, while the MICA-4 was used to determine the attitudes of the students.

The MAKS was developed in the United Kingdom (UK) to evaluate stigma-related MH knowledge (Evans-Lacko et al. 2010). Items on the MAKS were scored on a Likert scale with responses ranging from 1 (strongly disagree) to 5 (don’t know). The total score for stigma-related statements ranged from 6 to 30 with higher scores indicating less stigma-related knowledge (Martensson, Jacobsson & Engstrom 2014). The MAKS has been shown to have a moderate to substantial overall test–retest reliability of 0.71 Lin’s concordance statistic and an overall internal consistency of 0.65 (Cronbach’s α) (Evans-Lacko et al. 2010). According to the authors’ knowledge this scale has not been used in South Africa.

The self-administered MICA-4 scale assesses attitudes towards psychiatry and people with mental illness. The MICA-4 scale is used by nurses and other professionals in health and social services (Siddiqua & Foster 2015). We used the MICA-4 because third and fourth-year physiotherapy students have some clinical experience as these are their clinical placement years. The MICA-4 scale consists of 16 questions and responses are also scored using a Likert-type scale. The individual item scores are added together. Scores range from 16 to 96 with an overall low score indicating a more positive attitude. The internal consistency of the MICA is 0.79 (Cronbach’s α) with a split-half correlation of 0.73. The test–retest reliability is high at 0.80. Weekly reminders to complete the surveys were sent to the students via emails sent through the year coordinators.

### Statistical analysis

The data were entered onto Microsoft Excel and analysed by the Statistical Package for Social Sciences (SPSS version 27) with assistance from a statistician at the University of the Witwatersrand. The following statistical tests were utilised: means, standard deviations, frequencies and proportions to summarise demographic data. Means and standard deviations were used to summarise the sum of the knowledge and attitude scores. A multiple linear regression was undertaken to determine differences in knowledge and attitudes between third- and fourth-year students.

### Ethical considerations

Permission to conduct our study was requested from the head of the Department of Physiotherapy at the University of the Witwatersrand, the head of the School of Therapeutic Sciences and the university registrar as students were the participants. Ethical clearance was obtained from the Human Research Ethics Committee (Medical) (Ethics clearance number: M200913). Completing the survey questions implied consent was given by the participant.

### Pilot study

The survey (demographic section, MAKS and MICA) was loaded onto REDCap and sent to 10 students who satisfied the inclusion criteria. There were only five responses received. The aim of our pilot study was to assess whether the questions were easy to understand, respond to and to determine the estimated time taken to answer the questions. A feedback section was included in the survey to allow the participants to comment on the questionnaire and alterations were made accordingly. Some feedback received reported that some of the statements were vague and as such the student was unsure how to answer the question. The only change made was the addition of examples to make the statements less vague.

## Results

### Demographic profile of physiotherapy students

A total of 11 third year and 23 fourth year students completed the online questionnaire. The total number of students in third and fourth years during 2021 was 111. This means that 34 (31%) of the students responded to the online questionnaire. Two-thirds of the participants were fourth year students with a mean age of 21 years. Of these students, 52.9% were female. Only one student expressed having prior experience with MHD (Table 1).

**TABLE 1:** Demographic profile of physiotherapy students (*n* = 34).

Variable	*n*	%	Average	s.d.
**Gender**				
Male	16	47.10	-	-
Female	18	52.90	-	-
Age	-	-	21.74	2.25
**Year of study**				
Third year	11	32.40	-	-
Fourth year	23	67.60	-	-
**Ethnicity**				
Black students	13	38.24	-	-
White students	13	38.24	-	-
Mixed race students	3	8.82	-	-
Indian students	5	14.71	-	-
Asian students	0	0.00	-	-
**Prior experience working in a mental health facility**				
Yes	1	2.90	-	-
No	33	97.10	-	-

s.d., standard deviation.

### Knowledge of the roles of physiotherapy in mental health

The average total MAKS score was 26.94 (standard deviation [s.d.]: 3.57), and the highest possible score was 36. This score indicated moderate knowledge of MHD. Most participants correctly identified that depression was a type of MHD, together with schizophrenia and bipolar disorder (Table 2). Furthermore, only 50% of the participants agreed that drug or substance use was a type of MHD (Table 2).

**TABLE 2:** Scores from the Mental Health Knowledge Schedule questionnaire.

MAKS question	Frequency (%) (*n* = 34)					
	Agree strongly	Agree slightly	Neither agree nor disagree	Disagree strongly	Disagree slightly	Don’t know
1. Most people with mental health problems want to have paid employment	32.35	32.35	14.71	2.94	5.88	11.80
2. If a friend had a mental health problem, I know what advice to give them to get professional help	23.53	58.82	11.77	2.94	2.94	23.53
3. Medication can be an effective treatment for people with mental health problems	32.40	44.10	14.70	0.00	5.90	2.90
4. Psychotherapy (e.g. talking therapy or counseling) can be an effective treatment for people with mental health problems	55.90	41.20	0.00	0.00	2.90	0.00
5. People with severe mental health problems can fully recover	20.60	29.40	17.60	8.80	11.80	11.80
6. Most people with mental health problems go to a healthcare professional to get help	5.90	8.80	8.80	47.10	26.50	2.90
**Do you think each condition is a type of mental illness? Check 1 box only**						
7. Depression	61.77	23.53	2.94	8.82	2.94	0.00
8. Stress	35.30	23.53	8.82	17.70	8.82	5.98
9. Schizophrenia	73.50	11.80	5.90	2.90	0.00	5.90
10. Bipolar disorder (manic depression)	79.40	8.82	0.00	8.82	0.00	2.92
11. Drug/substance addiction	50.00	20.60	5.90	11.80	2.90	8.80
12. Grief	8.82	35.30	14.71	14.71	23.53	2.94
**The following are roles of physiotherapy in mental health**						
13. Informing individuals adequately about mental health	58.82	32.35	2.94	2.94	2.94	0.00
14. Eliminate misconceptions about mental illness	82.40	11.80	2.90	0.00	2.90	0.00
15. Refer necessary patients to specialised professionals in mental health and psychiatry	85.30	5.90	0.00	0.00	5.90	2.90
16. Optimising well-being	91.18	2.94	0.00	2.94	2.94	0.00
17. Empowering the individual by promoting functional movement	82.40	8.80	2.90	0.00	5.90	0.00
18. Promoting movement awareness	82.40	8.80	2.90	0.00	5.90	0.00
19. Promoting physical activity and exercises	88.20	5.90	0.00	0.00	5.90	0.00

*Source:* Adapted from Evans-Lacko, S., Little, K., Meltzer, H, Rose, D., Rhydderch, D., Henderson, C., 2010, ‘Development and psychometric properties of the mental health knowledge schedule’, *Canadian Journal of Psychiatry* 55(7), 440–448. https://doi.org/10.1177/070674371005500707.

MAKS, Mental Health Knowledge Schedule.

### Attitudes about the role of physiotherapy in mental health

The total number of participants who completed the MICA-4 questionnaire completely was 30. The average score was 39.12 (s.d.: 16.12), the highest possible score was 96. A score of 39.12 showed a positive attitude. Four questionnaires were excluded from the analysis as they had incomplete information as not all questions were answered and therefore could not be included in the analysis.

**FIGURE 1:**
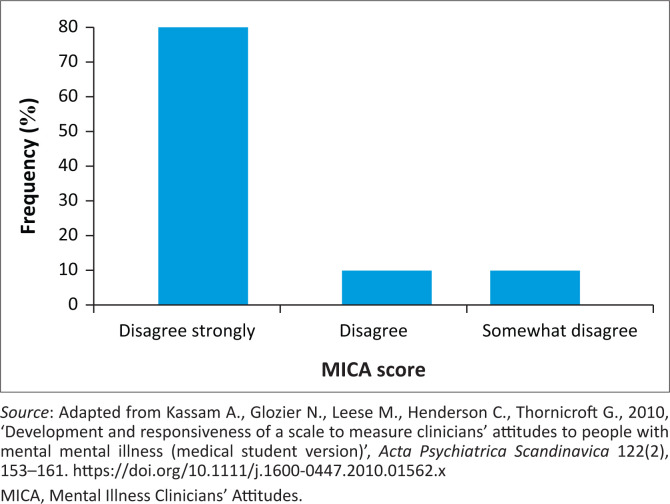
Scores of the question, ‘I would use the terms “crazy,” “nutter,” “mad” to describe to colleagues people with a mental illness that I have seen in my work’.

**FIGURE 2:**
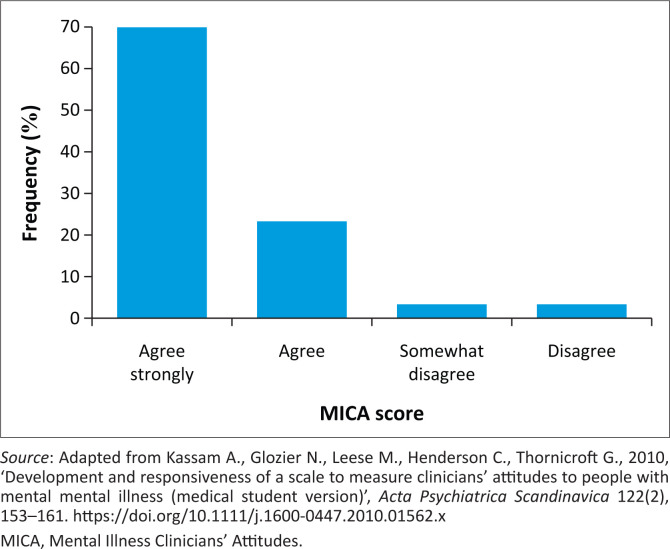
Scores of the question, ‘Working in the mental health field is just as respectable as other fields of health and social care’.

**FIGURE 3:**
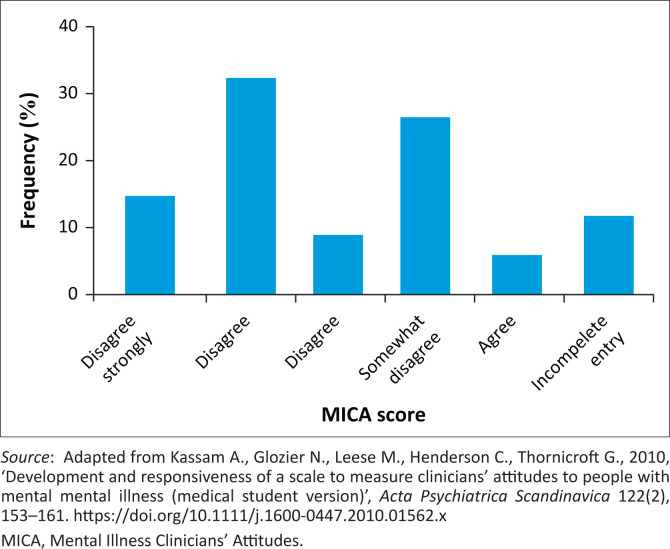
Scores for the question, ‘I just learn about mental health when I have to and would not bother to read additional material on it’.

Eighty percent (*n* = 24) strongly disagreed that they would use terms like ‘nutter’, ‘crazy’ or ‘mad’ to describe people with mental illness. Also, 70% (*n* = 21) of respondents ‘agreed’ that working in mental health was just as reputable as working in other professions; however, 32.4% reported that they would not learn about MHD if they did not have to (see Figure 1, Figure 2 and Figure 3). Half the students (53.3%) ‘strongly disagreed’ that healthcare professions working with patients with mental illness should also ensure that they get the patient’s physical health assessed. Likewise, half the students (53.3%) ‘disagreed’ that being a healthcare professional working in the mental health field was not the same as a ‘real’ healthcare professional.

Almost an equal proportion of students ‘disagreed’ (26.7% ‘strongly disagreed’ and 30% ‘disagreed’) that they would never admit having mental illness to their friends for fear of being treated differently. Most students ‘disagreed’ that general practitioners should not do a thorough assessment for people with psychiatric symptoms because they can be referred to a psychiatrist (30% ‘strongly disagreed’, 26.7% ‘disagreed’ and 26.7% ‘somewhat disagreed’).

A variable pattern with almost equal percentages across the responses was observed with the statement ‘Health/social care professionals know more about the lives of people treated for mental illness than do family members or friends’. For this statement, 26.7% of the participants ‘disagree’ and 26.7% ‘somewhat agree’, 16.7% ‘disagree strongly’, 10% ‘somewhat disagree’, 10% ‘agree’ and 10% ‘agree strongly’ for each response.

### Differences in knowledge and attitudes between third- and fourth-year physiotherapy students

The multiple linear regression determined differences in knowledge and attitudes between third- and fourth-year physiotherapy students. The fourth-year students had a lower MAKS score (26.70) compared to their third-year counterparts (27.45) which demonstrated more knowledge in the fourth-year students. The third-year students had a higher MICA score (40.09) compared to fourth-year students (38.65) (Table 3). This indicated that third-year students had a more negative attitude towards mental illness compared to their fourth-year counterparts. These differences in knowledge and attitude scores were not statistically significant. When considering a relationship between the MICA and MAKS scores between the two year groups the multiple linear regression coefficients indicated no statistically significant relationship between either MICA (*p* = 0.907; β = 0.022) or MAKS (*p* = 0.570; β = 0.101). The multiple linear regression relationship between year groups and gender also revealed no statistically significant differences for MICA (*p* = 0.624; β = 0.093) and MAKS (*p* = 0.846; β = 0.035).

**TABLE 3:** Differences in the attitude (Mental Illness Clinicians’ Attitudes-4) scores between the year groups.

Year of study	Gender	Average MICA-4 score	Standard deviation
Third	Male	44.00	9.03
	Female	36.83	18.90
Total for third years		40.09	15.00
Fourth	Male	37.36	20.07
	Female	39.83	14.31
Total for fourth years		38.65	16.94

MICA, Mental Illness Clinicians’ Attitudes.

## Discussion

Our study aimed to determine the knowledge and attitudes that physiotherapy students at the University of the Witwatersrand have towards the role of physiotherapy in mental health. The results demonstrated that third- and fourth-year physiotherapy students have some knowledge of the role of physiotherapy in mental health. Additionally, a good attitude towards mental illness was observed. Third-year physiotherapy students had less knowledge and a more negative attitude towards mental health than their fourth-year peers. This finding is similar to that of Probst and Peuskens (2010), who found that students who were more familiar with mental illness had more positive attitudes about mental illness than students without previous exposure.

Knowledge can be defined as factual information acquired through learning (Geer et al. 2006). As was seen in the results, where most participants could identify depression, schizophrenia and bipolar disorder as MHD types some did not recognise drug or substance abuse as a type of MHD. The study conducted by Vancampfort et al. (2018) showed that drug and substance abuse makes up almost a fifth of all disability-associated burdens in sub-Saharan Africa. With the high prevalence of depression and substance abuse, particularly in South Africa, students are more likely to attend to patients with these disorders in their clinical placements and after graduating. Knowledge of these more common MHD enables the physiotherapist to screen for substance abuse, educate patients and refer to appropriate health care professionals such as psychiatrists and psychologists for diagnosis and management.

Limited knowledge contributes to physiotherapists feeling ill-equipped to manage patients with MHD (Driver et al. 2017; Hooblaul, Cobbing & Daniels 2020). Physiotherapists in Kwazulu-Natal, South Africa, reported a need for more comprehensive training to improve their knowledge of people living with MHD (Hooblaul et al. 2020) in both undergraduate and postgraduate training. Similarly, Dandridge et al. (2014) reported the majority of physiotherapy students in the UK (75%) expressed a need for further training in mental health with most (71%) having less than 4 h of training in mental health. This training was often within a broader module or within a single lecture. The preferred method of training was in the form of clinical placements and seminars (Dandridge et al. 2014).

The differences in knowledge and attitudes to MHD between student year groups could be linked to differences in clinical exposure. Clinical placements and seminars afford student physiotherapists exposure to patients with MHD. The survey used in our study was sent out between March 2021 and October 2021 which gave sufficient time for the students to go on at least one clinical placement. Fourth-year students have more clinical exposure as they are further in their degrees thus may have more experience in the management of patients with MHD. Third year students are only being exposed to clinical placements for the first time so their general knowledge, skills and clinical reasoning may be less developed. Additionally, fourth-year students received a 1.5-h online lecture on MHD before commencing their clinical placements, whereas the third-year students did not. This was delivered as an online recording so they could access the information again as needed in placements.

Physiotherapists have a recognised role to play in the management of MHD. Most of the participants recognised that physiotherapists contribute to managing patients with MHD by optimising well-being, promoting physical activity and referral to specialised professionals in mental health and psychiatry. Appreciating this role among physiotherapy students is critical as they can contribute effectively to the mental health MDT and improve patients’ quality of life. Additionally, these roles are similar to other studies which state that the physiotherapist is responsible for encouraging movement and function, thus maximising an individual’s health (Probst 2017; Stubbs et al. 2014; Vancampfort et al. 2018).

Attitude can be defined as an emotion, feeling or desire for learning (Geer et al. 2006). There is a relationship between attitudes of health professionals towards people with MHD and subsequent treatment and interactions (Dandridge et al. 2014; Hansson et al. 2013). As stated previously, fourth-year physiotherapy students had a more favourable attitude towards mental illness compared to the third-year students, which is like the study by Probst and Peuskens (2010). Additionally, Probst and Peuskens (2010) demonstrated that students had a better attitude towards patients with psychiatric conditions after receiving training in these conditions, suggesting a link between knowledge and attitudes, which is similar to our findings. In our study, third-year students had higher MAKS and MICA-4 scores which suggested less knowledge and a more negative attitude compared to their fourth-year counterparts. Furthermore, fourth-year students had a more favourable attitude towards MHD. A negative attitude towards patients with MHD could lead to bias and affect the quality of physiotherapy care, thus influencing patient adherence to physiotherapy and patient satisfaction (Probst & Peuskens 2010).

### Strengths and limitations of study

Our study had some limitations. Firstly, it was conducted using online questionnaires on REDCap. The response rate may have been higher if the surveys were completed in a face-to-face environment; however, this was not possible as our study was conducted during the coronavirus disease 2019 (COVID-19) pandemic. Secondly, there was a poor response rate of 34% and therefore results are not generalisable. Despite regular emails to remind students to participate and invitations to participate being sent through other digital communication platforms, the level of response remained low. This could have been attributed to busy schedules or being asked to participate in too many surveys. Thirdly, a cross-sectional design was used; hence, a control group was not included with which to make comparisons. Another limitation is that the MAKS and MICA-4 scales are tailored specifically for ‘allied’ health professionals who have experience working with patients with MHD. For example, a question in the MAKS survey such as ‘Most people with mental health problems go to a healthcare professional to get help’ would be easier to answer if the responder had prior experience working with these patients. Also, both surveys applied to qualified physiotherapists and not students.

A strength of our study was the fact it was the first of its kind at the University of the Witwatersrand. The surveys gave some useful information on the knowledge of the roles of physiotherapy in mental health and identifying common types of MHD.

Future studies could consider reviewing different teaching models and the effect they may have on knowledge and attitudes towards MHD.

## Conclusion

Our study aimed to establish the knowledge and attitudes that physiotherapy students at the University of the Witwatersrand have regarding the role of physiotherapy in mental health. It was limited to the third- and fourth-year physiotherapy students and a response rate of 31% was attained. The low response rate was a limitation of our study and reduced the generalisability of the findings. Results showed that third-year students had less knowledge and a more negative attitude about MH compared to their fourth-year counterparts. The results suggest that the more the students are taught and exposed to MHD, the more likely their attitude and their understanding of their role in MH improves. Our study should be expanded to consider all the years of study as well as all universities offering physiotherapy degrees in South Africa.
